# Magnitude and Determinant Factors of Herbal Medicine Utilization Among Mothers Attending Their Antenatal Care at Public Health Institutions in Debre Berhan Town, Ethiopia

**DOI:** 10.3389/fpubh.2022.883053

**Published:** 2022-04-29

**Authors:** Getu Engida Wake, Girma Wogie Fitie

**Affiliations:** Department of Midwifery, Institute of Medicine and Health Science, Debre Berhan University, Debre Berhan, Ethiopia

**Keywords:** herbal, traditional medicine, utilization, Ethiopia, mothers

## Abstract

**Background:**

World health organization defined herbal medicines as the practice of herbs, herbal materials, herbal preparations, and finished herbal products. Globally women are the primary utilizer of herbal medicine and even they consume it during their pregnancy period. The World health organization reported that the majority of the global population used traditional medicine. This study aimed to assess the magnitude and determinant factors of herbal medicine utilization among pregnant mothers attending their antenatal care at public health institutions in Debre Berhan town, Ethiopia.

**Methodology:**

Institution-based cross-sectional study was conducted among pregnant mothers who attended their antenatal care at public health institutions in Debre Berhan town, Ethiopia from 12 February 2021 to 12 April 2021. A systematic random sampling method was used to get selected pregnant mothers. Epi data version 3.1 and SPSS version 25 were used for data entry and analysis, respectively. *P*-value ≤0.05 was used as a cut point of statistical significance in multivariable binary logistic regression.

**Results:**

A total of 422 pregnant mothers were included with a 100% response rate and 277 (65.6%) of them used an herbal medicine during their current pregnancy. Educational level up to primary school [AOR 2.21, 95% CI: 1.17 – 4.18], monthly family income of <2,800 ETB [AOR 1.72, 95% CI: 1.01–2.92], and lack of awareness of the complication of herbal medicine utilization [AOR 10.3, 95% CI: 6.27–16.92] were determinant factors of herbal medicine utilization.

**Conclusion:**

The utilization of herbal medicine among pregnant mothers in this study is high and almost all of them did not disclose their utilization of herbal medicine to their healthcare providers. The ministry of health should integrate traditional medicine with conventional medicines. Midwives and obstetricians should openly discuss regarding benefits and complications of herbal medicine utilization for those pregnant mothers during their antenatal care counseling sessions as routine care.

## Introduction

Traditional medicine is defined as the ways of protecting and restoring health that existed before the arrival of modern medicine (1). It is underestimated part of healthcare that finds in almost every country in the world (2). Traditional medicine has been being used in the maintenance of health and the prevention, diagnosis, improvement, or treatment of physical and mental illness (3). According to the WHO, herbal medicine is defined as the practice of herbs, herbal materials, herbal preparations, and finished herbal products (4), and they are derived from plant parts such as leaves, stems, flowers, roots, and seeds (5). Globally in the previous decade, there has been revived need and interest in the use of traditional medicine (6). The WHO estimated that 80% of the global population used traditional and complementary medicine as primary healthcare (3). The utilization of traditional medicine has maintained its global popularity and it varies from country to country (1). In Asian countries, the consumption of traditional medicine ranges from 40% in China to 65% in India (1, 7). Similarly in European countries utilization of traditional medicine accounts for 31% in Belgium, 49% in France, and 70% in Canada (7).

Approximately 80% of the population in Africa used traditional medicine (6), and evidence indicated that in sub-Saharan African countries (SSA) the prevalence of traditional medicine utilization among pregnant mothers was between 25 and 65% (8). Even though there is insufficient data on the safety of herbal medicine utilization during pregnancy (9), local herbal products were being recommended by healthcare professionals in sub-Saharan African countries (SSA) for different health-related problems during pregnancy (10). Herbal medicines toxicity can be related to a lack of proper standardization, absence of quality control, and adulteration of herbal products with other pharmaceutical drugs and potentially toxic substances. Hence utilization of some unstudied herbal medicines with unknown pharmacologic activity can end up in adverse health outcomes for some vulnerable groups such as older adults, children, and pregnant women and their fetuses (11–13).

According to the results of some kinds of literature, overutilization of herbal medicine during pregnancy is associated with different maternal and child adverse health outcomes such as preterm birth, cesarean birth, low birth weight, vaginal bleeding during pregnancy, maternal and neonatal morbidity and mortality, different congenital anomalies such as cleft lip, hypoplastic left heart syndrome, inguinal hernia, hydronephrosis, duplicate renal pelvis, fetal ductus arteriosus constriction, trisomy 18, and different form of maternal gastrointestinal complaints (14, 15). Globally women are the primary utilizer of herbal medicine (HM), and even they consume different herbal medicine during the pregnancy period (16). The consumption of herbal medicine among pregnant and childbearing mothers ranges from 7 to 55% (17), and this difference depends on the consumer's geographic location, ethnicity, culture, traditions, and social status (16). Accordingly, utilization of herbal medicine among pregnant mothers was 34% in Australia (18), 50% in European Union (19, 20), and 6–9% in the USA and Canada, respectively (21, 22). Herbal products are believed a safe and natural alternative to conventional drugs among pregnant mothers and are used for the treatment of non-life treating conditions such as nausea and constipation (23). Globally, herbal medicine is available over the counter which makes them very accessible for utilization despite its health consequence when self-prescribed by pregnant women (24).

Many studies had revealed that pregnant women used different types of herbal medicine and the most commonly used herbal medicines were ginger (*Zingiber officinale* Roscoe), Chamomile (*Matricaria chamomilla* L.), peppermint (*Mentha* piperita L.), Echinacea (*Echinacea purpurea* L.), cranberry (*Vaccinium oxycoccus* L. and *Vaccinium macrocarpum* L.), garlic (*Allium sativum* L.), raspberry (*Rubus idaeus* L.), valerian (*Valeriana officinalis* L.), fenugreek (*Trigonella* foenum*-*graecum L.), fennel (*Foeniculum vulgare* Mill.), herbal blends, and teas, namely, green and black teas [*Camellia sinensis* (L.) Kuntze)(25–27). Pregnant mothers use herbal medicine for mother or child-health-related problems and the most commonly reported indications for utilization of herbal medicines were nausea, vomiting, urinary tract infections (UTIs), preparation or facilitation of labor, cold, gastrointestinal problems, improvement of fetal outcomes and prevention of miscarriage, anxiety, health maintenance, and edema (26, 27). Moreover, pregnant mothers consume herbal medicines due to their easy accessibility, assumed better efficacy compared to modern medicine, traditional/cultural belief, and low cost of herbal medicines compared to conventional medicine (28, 29).

Some evidence from Australia and Kenya showed that older and married pregnant mothers with low economic status, low educational level, and those who had nausea, and vomiting were the most utilizers of herbal medicine (29–32). Another literature has also found that herbal medicine use during pregnancy was determined by some factors such as higher maternal age, lower educational level of the spouse, poor pregnancy outcomes, previous herbal medicine utilization large family size, self-employment, unemployment, and rural residence in addition to previously mentioned factors (33).

Nearly 80% of the Ethiopian population uses traditional medicine (34). The consumption of herbal medicines in Ethiopia is not only common but also culturally accepted and acknowledged (35). Evidence indicated that the practice of herbal medicine in Ethiopia ranges from 40.6% in Harar to 73.6% in Hosanna (36, 37). The cultural acceptability of healers and local pharmacopeia, the relatively low cost of traditional medicine, and difficult access to modern health facilities were some of the reasons for herbal medicines utilization in Ethiopia (38). The majority of the pregnant mothers are unaware of the possible maternal and fetal complications of herbal medicine utilization (29, 39), and those pregnant mothers and breastfeeding women are vulnerable to harmful effects of herbal medicines consumption since the appropriate dosages of herbal medicines and safety are not well established (40). The study of prevalence and determinants of herbal medicine utilization among pregnant mothers is a current public health concern in many developing countries including Ethiopia. In addition, even though some studies were conducted in Ethiopia, there is a scarcity of data on the magnitude and determinants of herbal medicine utilization among pregnant women. Therefore, this study aimed to assess the magnitude and determinant factors of herbal medicine utilization among mothers attending their antenatal care visit at public health institutions in Debre Berhan town Ethiopia.

## Methods

### Study Design and Study Period

An institutional-based cross-sectional study was conducted from 12 February 2021 to 12 April 2021.

### Study Setting and Participants

The study was conducted in Debre Berhan town, which is one of the 13 zones of the Amhara regional state. Debre Berhan town is located 130 km to the north of Addis Ababa city. It is found at an altitude of 2,850 m from sea level with a temperature ranging from 13 to 28°C. The town has nine kebele (seven kebele has an urban population while two of the kebeles have both urban and rural populations). Regarding the number of health institutions, the town has one comprehensive referral hospital, two private hospitals, three public health centers, nine health posts, and 18 private clinics. Pregnant women who came to attend antenatal care at public health institutions in Debre Berhan town during the study period were our study population.

### Inclusion and Exclusion Criteria

Pregnant women who came for antenatal care visits at public health institutions in Debre Berhan town during the data collection period were included, while pregnant mothers who were seriously sick, who could not come to public health institutions and be unable to respond during the data collection time were excluded from the study.

### Sample Size Determination, Sampling Technique, and Procedure

The sample size was determined using a single population proportion formula based on the assumption of 95% CI, 5% margin of error, and 48.6% prevalence of herbal medicine utilization (41).


$$\begin{array}{l} N=\frac{\left(Z\;\alpha /2\right)2\;*P\;*\;\left(1-P\right)}{d2} \end{array}$$

Where;

*n* = the actual sample size

*Z* = the standard normal deviation at 95% CI; =1.96

*P* = proportion of herbal medicine utilization

*d* = margin of error that can be tolerated, 5% (0.05)

*n* = $\frac{\left(1.96\right)2\;*\;0.486\;*\;\left(1-\;0.486\right)}{\left(0.05\right)2}=$383.

By considering a 10% of non-response rate (*n* = 39, the final sample size become (*N* = 422) pregnant mothers.

There are a total of four public health institutions in Debre Berhan town that provide focused antenatal care and we included all four public health institutions. The numbers of pregnant mothers who visited the public health institutions which were surveyed from each health institution were allocated proportionally based on the expected number of pregnant mothers who visited the public health institutions for the study period and the estimation was made depending on the number of pregnant mothers who visited each health institution for the last 2 months. The proportional allocation was calculated using the following formula:

$$\begin{array}{l} nj=\;n/N\;*\;Nj \end{array}$$

Where:

*nj* = Sample size of the *j*th health institution

*n* = total sample size

*Nj* = number of pregnant mothers who visited the *j*th health institution in the last 2 months.

**Figure 1:**
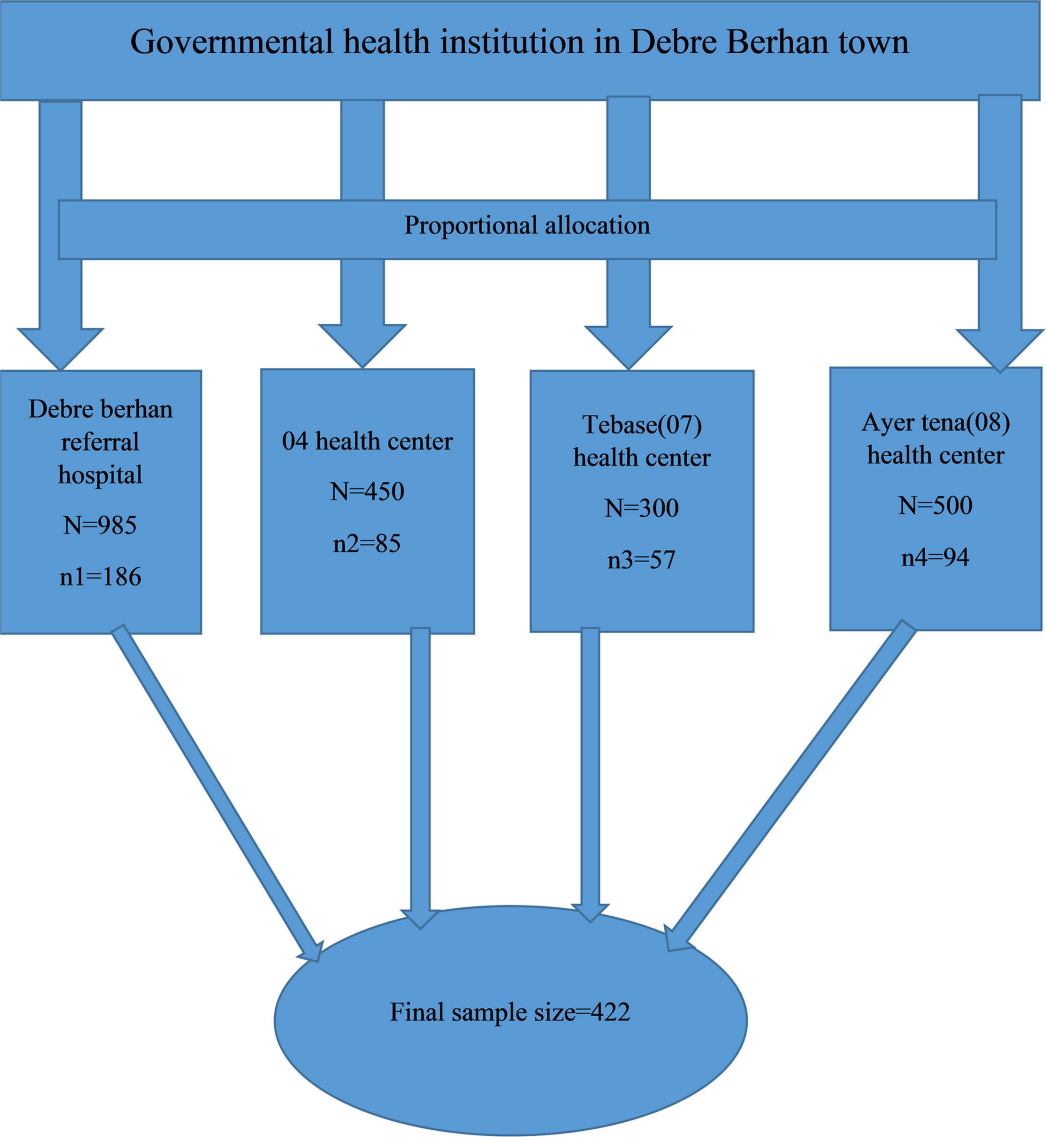
Schematic presentation of sampling procedure of the study.

*N* = Total number of pregnant mothers who visited all public health institutions in the last 2 months. Lastly, study participants were selected systematically (*k* = 5) based on the order of pregnant mothers who come to antenatal care rooms at health institutions until the required sample size was obtained *K* = 2235/422 = 5^th^ (Figure 1).

### Operational and Definition of Terms

➢ Herbal medicine use: refers to using the seeds, berries, roots, leaves, bark, or flowers of a plant for medicinal purposes.
➢ Herbal medicine utilization in current pregnancy: respondents were labeled as herbal medicine users if they have taken herbal medicine *via* any route of administration during the current pregnancy period. Routine meal preparations and nutrients such as food additives were excluded.
➢ Knowledge was measured using four items prepared to assess it. Study participants were asked the knowledge-related questions and value one was given for correct answers and value zero was given for those incorrect answers. Then respondent's score was dichotomized as sufficient knowledge or insufficient knowledge after the total score was computed by summing up all the items together.
➢ Sufficient in knowledge: Study participants who answered equal to or greater than the mean values of knowledge-related questions.
➢ Insufficient knowledge: Study participants who answered less than the mean values of knowledge-related questions.

### Methods of Data Collection Tool, Procedure, and Quality Control

Data were collected by face-to-face interviews administered using a semi-structured questionnaire. Five-degree pharmacy and two adult nursing masters were recruited as data collectors and supervisors, respectively. The data collection tool was developed from different published literature and slight modification was made to the questions to make them in line with the objective of our study (35–37, 41). The questionnaires were designed in the English language, translated into Amharic, and back to the English language for consistency of collected data. Twenty-six items were included in the final questionnaire divided into three sections. The first section covered data regarding sociodemographic and pregnancy-related information such as age, marital status, ethnicity, educational status of the mother, employment status, religion, monthly income, parity, presence or absence of ANC visiting history, presence or absence of health problems not related to gestation, trimester of pregnancy and distance from the health facility. The second section aimed at assessing the knowledge level of herbal medicine among pregnant mothers and it was assessed by a series of questions such as whether they have heard about herbal medicine or not, the types of herbal medicine they knew, information about the complication of herbal medicine utilization and types of complications of herbal medicine utilization they knew. The third section was used to collect data concerning the level of herbal medicine utilization among pregnant mothers, source of information regarding herbal medicine, presence or absence of discussion with their healthcare providers about herbal medicine utilization, and satisfaction level of pregnant mothers toward utilization of herbal medicine. The utilization of herbal medicine among pregnant mothers was assessed by different questions such as the utilization of herbal medicines during pregnancy, reason of use among herbal medicine utilizer, type of herbal medicine used, the purpose of herbal medicine utilization, trimester of herbal medicine utilization, source of information about herbal medicine use and any untoward effects faced during their utilization of herbal medicines.

To maintain data quality, data collectors were given training for 2 days about the overall research objective including data collection procedures, tools, and how to fill data. In addition, the questionnaire was pretested in 10% of the sample size in Ataye hospital 3 weeks before the actual data collection period, and necessary amendments such as language clarity and appropriateness of the tools were done based on the findings of the pretest before the actual data collection time. Collected data were reviewed and checked for completeness and consistency by supervisors and the principal investigator daily.

### Methods of Data Entry and Analysis

The collected data was cleaned, coded, and entered into Epidata version 3.1 and exported to statistical package for social science (SPSS) version 25 for analysis. Bivariable logistic regression was used to identify the determinant factors of herbal medicine utilization among pregnant mothers. Variables with a significant association in the bivariable analysis were entered into a multivariable binary logistic regression analysis to assess the determinant factors of herbal medicine utilization among pregnant mothers and *P-*values <0.2 and 0.05 were considered statistically significant for bivariable and multivariable binary logistic regression, respectively. The overall results were presented in texts, tables, and figures.

## Results

### Sociodemographic Characteristics of Study Participants

A total of 422 pregnant mothers were involved with a response rate of 100%. The mean age and average monthly family income of the study participants were 28 years old and 3,264 Ethiopian Birr (ETB), respectively. Almost all of them were Amhara and the majority of them were Orthodox religious followers (Table 1).

**Table 1:** Distribution of study participants by sociodemographic characteristics and their respective chi-square test at public health institutions in Debre Berhan town, Ethiopia, 2021 (*n* = 422).

**Variable**	**Category**	**Herbal medicine utilization**				**chi-square (*X*^2^)**	***p*-value**
		**No**		**Yes**			
		**Frequency**	**Percent**	**Frequency**	**Percent**		
Age	<20	12	8.3	31	11.2	1.06	0.59
	20–30	96	66.2	173	62.5		
	>30	37	25.5	73	26.4		
Religion	Orthodox	104	71.7	225	81.2	5.74	0.06
	Muslim	29	20	32	16.6		
	Protestant	12	8.3	20	7.2		
Ethnicity	Amhara	139	95.9	261	94.2	0.55	0.76
	Oromo	5	3.4	13	4.7		
	Tigre	1	0.7	3	1.1		
Marital status	Married	125	86.2	240	86.6	0.98	0.81
	Single	12	8.3	26	9.4		
	Divorced	4	2.8	7	2.5		
	Windrowed	4	2.8	4	1.4		
Educational status	Illiterate	12	8.3	36	13.0	13.896	0.003
	Primary school	18	12.4	66	23.8		
	Secondary school	47	32.4	85	30.7		
	College and above	68	46.9	90	32.5		
Employment status	Government employee	49	33.8	86	31.0		
	Self-employee	63	43.4	138	49.8	1.65	0.44
	Unemployed	33	22.8	53	19.1		
Average monthly income	<2800	52	35.9	155	56.0	16.01	0.001
	2800–4800	44	30.3	52	18.8		
	>4800	49	33.8	70	25.3		
Parity	Null	72	49.65	124	44.76	3.06	0.22
	1–2 children	56	38.62	103	37.18		
	3–4 children	17	11.72	50	18.05		
The complication of herbal medicine utilization	no	44	30.3	228	82.3	112.18	0.001
	yes	101	69.7	49	17.3		

### Knowledge and Practice of Pregnant Mothers Toward Herbal Medicine Utilization

**Figure 2:**
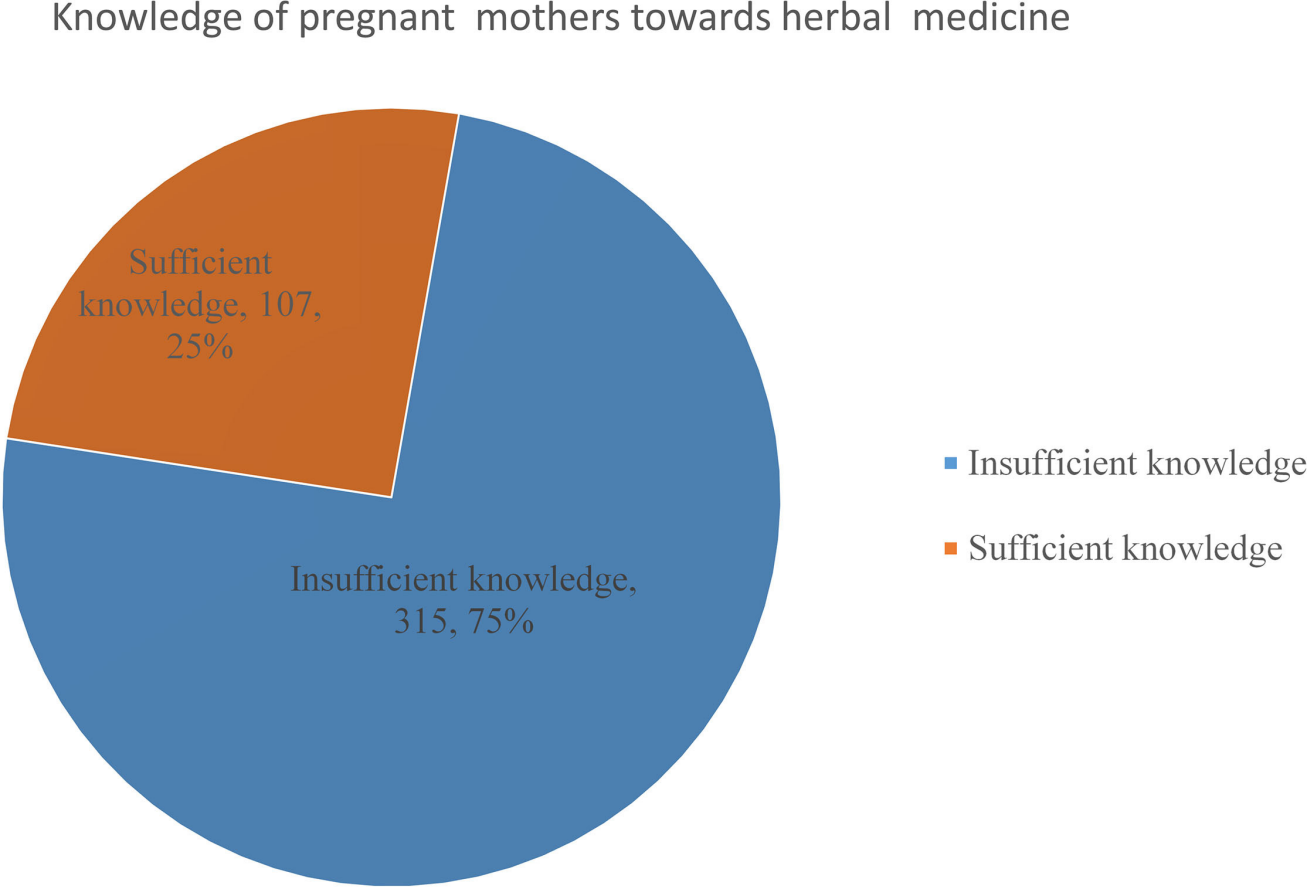
Knowledge of pregnant mothers toward herbal medicine among mothers who visited governmental health institutions for antenatal care in Debre Behan town, Ethiopia.

Of the total study participants, 420 (99.5%) respondents heard about herbal medicine from different sources and 150 (35.5%) knew about complications of herbal medicine utilization (Figure 2).

Of the total study participants, more than half of them used an herbal medicine during their current pregnancy. Among study participants who used herbal medicine in the current pregnancy, 163 used it during the first trimester of pregnancy. The most common source of information about herbal medicine was families followed by media (Table 2).

**Table 2:** Study participant's characteristics toward herbal medicine utilization at public health institutions in Debre Berhan town, Ethiopia, 2021 (*n* = 422).

**Variables**	**Frequency**	**Percent (%)**
**Herbal medicine use during the current pregnancy**		
No Yes	145 277	34.4 65.6
**Source of information of herbal medicine (*****n*** **=** **277)**		
Family members Healthcare professionals Media (internet, television, radio, book)	182 19 76	65.7 6.9 27.4
**Trimester**		
First trimester Second trimester Third trimester	163 88 26	58.8 31.8 9.4
**Reasons for herbal medicine use (*****n*** **=** **277)**		
Family, tradition, or culture Belief in the effectiveness of herbal medicines Herbal medicines are cheap and accessible Treatment of other medical problems	177 131 115 16	63.9 47.3 41.5 5.8
**Reason for not using herbal medicines among non-users (*****n*** **=** **145)**		
Lack of belief in the benefits of herbs Afraid the side effect Didn't get sick during gestation	22 60 63	15.2 41.4 43.4
**Discuss with HCPs about herbal medicine use (*****n*** **=** **422)**		
No Yes	397 25	94.1 5.9
**Side effects from herbal medicine use (*****n*** **=** **277)**		
No Yes	218 59	78.7 21.3
**Satisfaction with herbal medicine use (*****n*** **=** **277)**		
Very satisfied Partially satisfied Not satisfied	60 152 65	21.7 54.9 23.5

*HCPs, healthcare professionals*.

#### Types and Indications of Herbal Medicine Used During the Current Pregnancy

**Figure 3:**
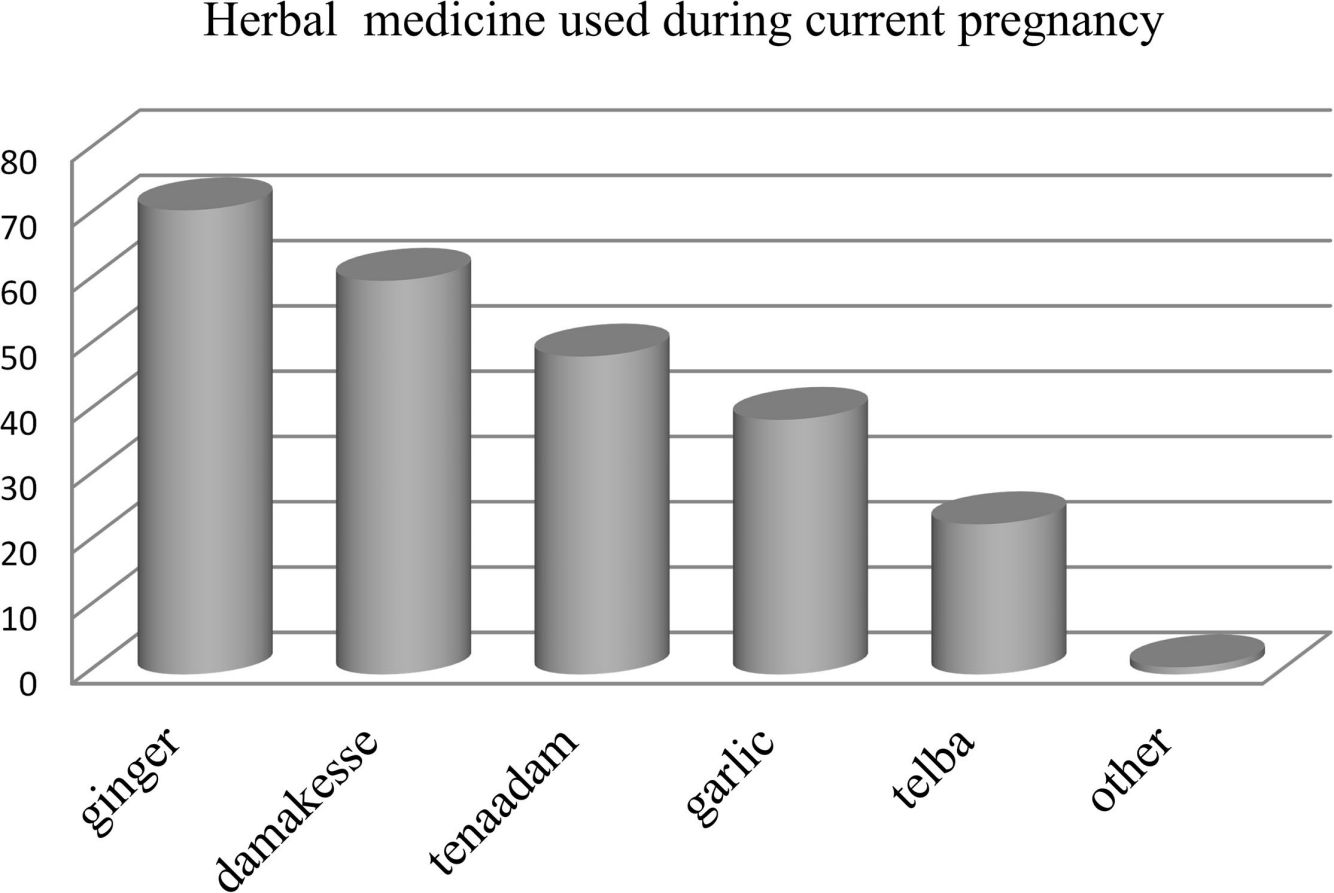
Type's herbal medicine used during current pregnancy at governmental health institutions in Debre Brehan town, Ethiopia.

**Figure 4:**
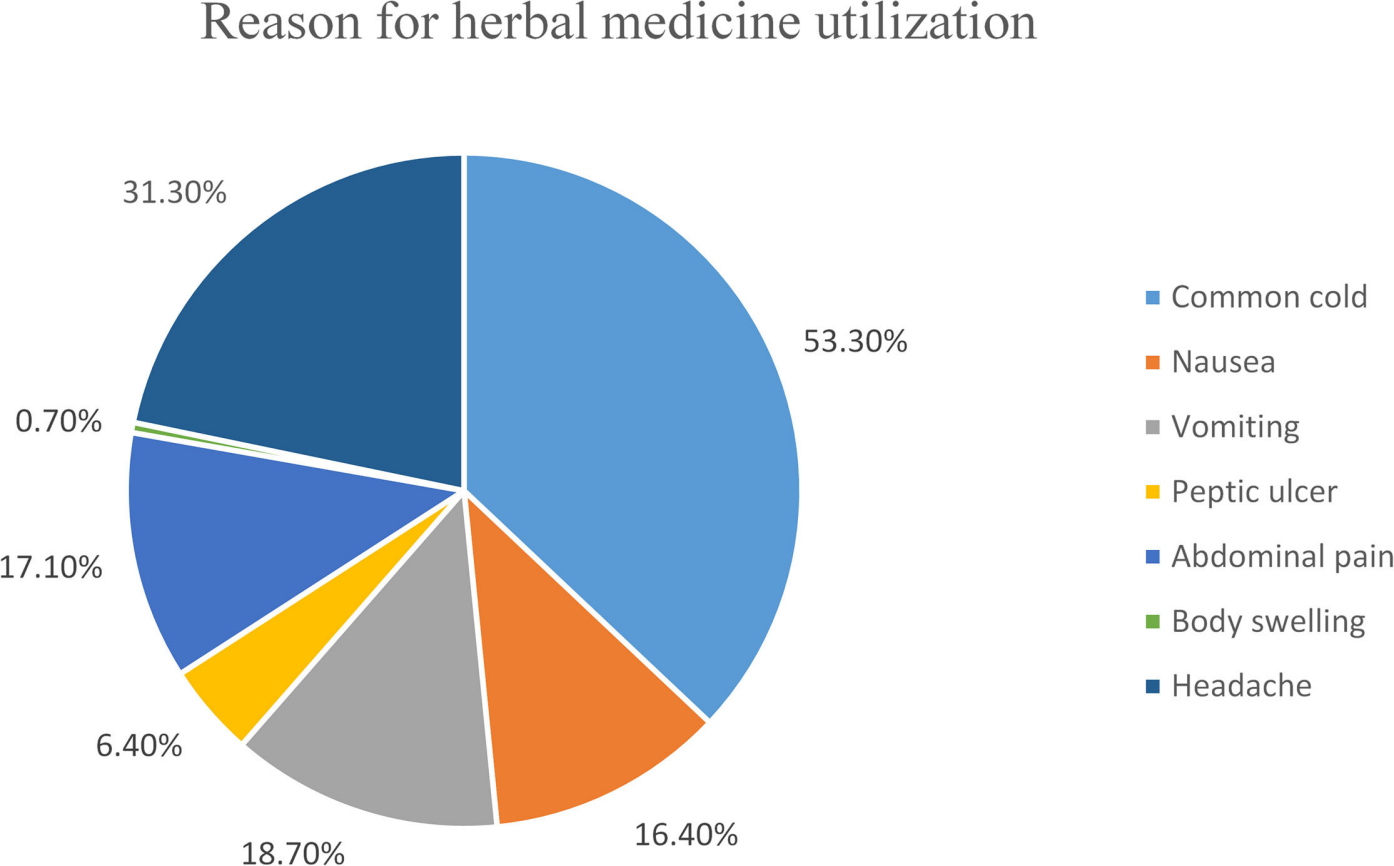
Indications for using the herbal medicine during the current pregnancy, among mothers who visited governmental health institutions for antenatal care in Debre Behan town, Ethiopia.

Of all respondents who stated that they had used an herbal medicine during their current pregnancy, the most commonly used herbal medicines were Ginger (*Zingiber officinale* Roscoe), Damakesse (*Ocimum lamiifolium*) followed by Tenadam (Fringed rue) (Figure 3). The common indications for the utilization of herbal medicine during current pregnancy were common cold and headache (Figure 4).

### Factors Associated With Herbal Medicine Utilization Among Pregnant Mothers

Bivariable and multivariable binary logistic regression were conducted to examine the determinant factors of herbal medicine utilization among pregnant mothers. In bivariable logistic regression variables such as educational level of pregnant mothers, average monthly income of the family, absence of ANC visit, presence of health problems not related to gestation, lack of discussion of herbal medicine utilization with healthcare professionals, knowledge level of pregnant mothers toward herbal medicine, and lack of awareness of complication of herbal medicine utilization were significantly associated with pregnant mothers herbal medicine utilization. But in multivariable binary logistic regression, only three variables (educational level, average monthly family income, and absence of awareness of complications of herbal medicine utilization) were significantly associated with the practice of herbal medicine among pregnant mothers. Thus, those pregnant mothers whose educational level was till primary school were 2 times more likely to consume herbal medicine during current pregnancy in comparison to study participants whose educational level was college and above [AOR: 2.21, 95% CI:1.17–4.18]. Those study participants who had a monthly family income of <2,800 Ethiopian Birr (ETB) were almost 2 times more likely to use herbal medicine during current pregnancy compared to those pregnant mothers who had a monthly family income of >4,200 Ethiopian Birr (ETB) [AOR: 1.72, 95% CI: 1.01–2.92]. Moreover, pregnant mothers who lacked awareness of complications of herbal medicine utilization were 10 times more likely to use herbal medicine during their pregnancy in comparison to studying participants who had awareness of complications of herbal medicine utilization [AOR: 10.3, 95% CI: 6.27–16.92] (Table 3).

**Table 3:** Bivariable and multivariable binary logistic regression analysis for factors associated with utilization of herbal medicine among pregnant mothers at public health institutions in Debre Berhan town, Ethiopia, 2021 (*n* = 422).

**Variables**	**Using herbal medicine**				
	**Yes**	**No**	**COR(95%CI)**	**AOR(95%CI)**	***P*-value**
**Educational status**					
Illiterate	36	12	2.27(1.10,4.68)	1.72(0.79, 3.74)	0.169
Primary school	66	18	2.77(1.51, 5.09)	2.21(1.17, 4.18)^*^	≤ 0.015^*^
Secondary school	85	47	1.37(0.85, 2.19)	1.22(0.74, 2.02)	0.431
College & above	90	68	1	1	
**Average monthly family Income**					
<2800	155	52	2.09(1.28, 3.38)	1.72(1.01, 2.92)^*^	≤ 0.045^*^
2800–4200	52	44	0.83(0.48, 1.42)	0.78(0.44, 1.37)	0.381
>4200	70	49	1	1	
**Discuss with HCPs about HMU**					
No	257	140	0.46(0.17, 1.25)	0.43(0.15, 1.23)	0.115
Yes **Do you have ANC?** No Yes **Presence of health problems not related to gestation** No Yes	20 65 212 259 18	5 24 121 141 4	1 1.55(0.92, 2.59) 1 0.41(0.14, 1.23) 1	1 1.76(0.96, 3.23) 1 0.39(0.13, 1.21) 1	0.68 0.105
**Level of knowledge**					
Sufficient	62	45	1	1	
Insufficient **Awareness of HMU complication** No Yes	215 228 49	100 44 101	1.56(0.99, 0.45) 10.68(6.68–17.10) 1	1.55(0.97, 2.47) 10.3(6.27–16.92)* 1	0.070 ≤ 0.001^*^

*AOR, Adjusted odds ratio; HCPs, healthcare professions; HMU, herbal medicine user; COR, Crude odds ratio; CI, confidence interval*;

* *, significantly associated value*.

## Discussion

This study aimed to assess the utilization of herbal medicine and its determinant factors among mothers attending their antenatal care visit at public health institution in Debre Berhan town, Ethiopia. This study found that the prevalence of utilization of herbal medicine during the current pregnancy was 65.6%. This finding is in line with the results of a study conducted in Zimbabwe (69.5%) (42), and lower than the result of a study conducted in Hosanna, Ethiopia (73.1%) (37). Moreover, this finding is higher than the finding of a study conducted in Nekemte hospitals, Ethiopia (50.4%) (35), public hospital of Harar, Ethiopia (40.6%) (36), University Gondar referral and teaching hospital, Ethiopia (48.6%) (41). This difference might be associated with the difference in sample size and study setting (some of them were conducted only in hospitals and other was community-based study). Besides this difference might be related to cultural differences across the regions of the country. Again this finding is higher than the results of the study conducted in Iran (48.4%) (43), Uganda (20%) (44), Tanzania (10.9%) (45), and Ghana (52.7%) (46). This difference might be related to cultural/belief variations across the countries, geographical differences, accessibility and affordability of herbal medicines, and methodological deference of the study such as study design, sample size, study setting, and included population.

Our study indicated that the most commonly used herbal medicine during the current pregnancy was ginger (71.1%). This finding is consistent with the results of a study conducted in Alexandria Egypt (32), Nekemte hospital, Ethiopia (35), and the University of Gondar referral and teaching hospital, Ethiopia (41). The similarity of this finding with the finding of a study conducted in Ethiopia might be associated with socio-cultural similarity and easy accessibility of herbs (ginger) all over the regions of Ethiopia and the study population of our study was similar to the study conducted in Alexandria Egypt. But this finding is different from the finding of two studies conducted in Iran (43, 47) which indicated sour orange and Ammi as commonly used herbal medicine, respectively. This difference could be attributed to socio-cultural differences and differences in types of herbal availability across the countries. Our study reported that the most common indication for herbal medicine utilization during the current pregnancy was the common cold (53.30%). This finding is in line with the report of a study conducted in a public hospital in Harar, Ethiopia, and the University of Gondar referral and teaching hospital, Ethiopia, respectively (36, 41). But the current finding is different from the results of a study conducted in Iran (43) and Malaysia (48) which indicated promotion of fetal health and facilitation of labor as the common indication for herbal medicine utilization, respectively.

More than half (59%) of pregnant mothers used herbal medicine in the first trimester of pregnancy and this finding is in line with the results of a study conducted in Nekemte hospital, Ethiopia (35), University of Gondar referral and teaching hospital, Ethiopia (41), and Iran (47). This consistency could be because many minor complications of pregnancy take place at the early stage of pregnancy and pregnant mothers took those herbal medicines to alleviate those minor problems. But this finding is different from the study conducted in Iran (43) and Malaysia (48), and both studies reported that the majority of study participants used herbal medicine in the third trimester of pregnancy. In our study, only (6%) of pregnant women disclosed utilization of herbal medicine during current pregnancy to their healthcare providers. This finding is in line with the result of a study conducted at the University of Gondar referral and teaching hospital, Ethiopia (41) and northern Uganda (44). This similarity might be because of the fear of pregnant mothers that healthcare providers might disagree with the idea and practice of herbal medicine during pregnancy if they disclosed the information to their healthcare providers (49).

Those pregnant mothers with a primary educational level were two times more likely to use herbal medicine during current pregnancy as compared to those who had an educational status of college and above. This finding is in line with the result of studies conducted in Hosanna, Ethiopia, and the University of Gondar referral and teaching hospital, Ethiopia (37, 41). The possible explanation is that those educated pregnant mothers could have information about the efficacy of modern or conventional medicine over herbal medicine. Again those educated pregnant mothers might have better information about the bad consequences of herbal medicine utilization during pregnancy and tend to use less traditional medicine in comparison to their counterparts.

Those pregnant mothers who had low average monthly income were almost 2 times more likely to use herbal medicine during their current pregnancy compared to their counterparts. This finding is in line with the results of studies conducted in the University of Gondar referral and teaching hospital, Ethiopia (41), Tanzania (45), and Ghana (46). Probably those pregnant mothers who were found in the lower socioeconomic class could have not afforded the cost of modern medicine and herbal medicine was more accessible and affordable for them to utilize when health-related problems happened. Those pregnant mothers who had no awareness of complications of herbal medicine utilization were 10 times more likely to use herbal medicine in comparison to pregnant mothers who had awareness of complications of herbal medicine utilization during their pregnancy and which is the new finding of this study. Probably lack of adequate information or knowledge on the complication of herbal medicine utilization exposed those pregnant mothers to utilize herbal medicine during their pregnancy. According to different pieces of evidence, herbal medicine utilization in some of the sub-Saharan African countries was associated with cultural and religion (50, 51) which is different from our study result which indicated the absence of a significant association between herbal medicine utilization and culture and religion of study participants. This might be associated with the difference in the ratio of traditional healers and healthcare professionals to the population in some sub-Saharan African countries. The ratio of traditional healers to the population in sub-Saharan Africa is 1:500, whereas the ratio of medical doctors to the population is 1:40,000 (52). Contrary to the above, there were large numbers of health extension workers in the community of Ethiopia which created adequate awareness of herbal medicine utilization during pregnancy.

## Limitations of the Study

The cause and effect relationship of the predictor variables with the level of pregnant mother herbal medicine utilization was not determined because of the cross-sectional nature of the study design. Besides, the study did not address the attitude of pregnant mothers toward the utilization of herbal medicine and the study did not assess the amount of herbal medicine the mother used.

## Conclusion

The utilization of herbal medicine among pregnant mothers in this study was high. The most commonly used herbal medicines were ginger (*Zingiber officinale* Roscoe), Damakesse (*Ocimum lamiifolium*), and Tenadam (*Fringed rue*). Common cold and headache were the common indications for utilization of herbal medicine during the current pregnancy period. Furthermore, educational level, average monthly family income, and absence of awareness of the complication of herbal medicine utilization were determinant factors of herbal medicine utilization among pregnant mothers. Governmental and non-governmental health institutions should promote traditional medicine practitioners to work together with modern medicine practitioners. Healthcare providers should openly discuss and create awareness about the benefit and complications of herbal medicine utilization during pregnancy giving special attention to those pregnant mothers who had a low educational level, low monthly family income and for those pregnant mothers who had no awareness of the complication of herbal medicine utilization during their antenatal counseling session as routine care. Again we recommend further research to be conducted by addressing the experience of herbal medicine use among users and providers through qualitative approaches.

## Data Availability Statement

The original contributions presented in the study are included in the article/supplementary material, further inquiries can be directed to the corresponding author/s.

## Ethics Statement

Ethical clearance was obtained from the research and ethical review board of Debre Berhan University, institute of medicine and health science (Ref. No. IHRRCB-020/04/2021). The letter of permission from the ethical review board and midwifery department was submitted to all governmental health institutions in Debre Berhan town. Besides, a letter of permission which was obtained from all four governmental health institutions in Debre Berhan town was submitted to each health institution's maternal and child health unit department. Lastly, informed written consent was obtained from pregnant mothers before data collection.

## Author Contributions

GW and GF: conceptualization, formal analysis, writing-original draft preparation, writing-review and editing, and funding acquisition. GW: methodology, data curation, and visualization. GF: software and supervision. All authors contributed to the article and approved the submitted version.

## Conflict of Interest

The authors declare that the research was conducted in the absence of any commercial or financial relationships that could be construed as a potential conflict of interest.

## Publisher's Note

All claims expressed in this article are solely those of the authors and do not necessarily represent those of their affiliated organizations, or those of the publisher, the editors and the reviewers. Any product that may be evaluated in this article, or claim that may be made by its manufacturer, is not guaranteed or endorsed by the publisher.

## Abbreviations

ANC: antenatal care
AOR: adjusted odds ratio
CI: confidence interval
COR: crude odds ratio
ETB: Ethiopian Birr
HCP: healthcare professionals
HMs: herbal medicine
HMU: herbal medicine utilization
OR: odds ratio
SPSS: Statistical Package for Social Sciences
SSA: sub-Saharan African
TM: traditional medicine
USA: Unites State of America
WHO: world health organization.
